# Notes and News

**Published:** 1892-04-16

**Authors:** 


### Notes and News.

Collected and Communicated.

The Institution of Naval Architects are opening out such prospects to the large army of bad and indifferent travellers by sea that it will be sad indeed if they should not be realised. The vibration from the engines in torpedo boats has proved so unbearable that science has turned its attention to overcoming the disadvantage. Is Mr. Yarrow a bad traveller, and the only one, or why have the horrors of sea sickness, so often directly traceable to the vibration, noise, and oil of machinery, been unfought before? The treatment by symptom has lamentably failed in this case, and we are glad to hear at least one cause of the malady is likely to be attacked.

During the past winter much horror and surprise has been felt at the numerous deaths from actual starvation which have been reported in our newspapers. In spite of the large charity organisations and the vast amount of money spent in charitable relief, we, with increased certainty, point to the lesson we should all learn, which is that the poor do not want more of our money, but they want our time and thoughts. Beggars are not those who die of starvation. Begging is a profitable trade because it appeals to the superficial pity of the public, and enables it to satisfy its conscience in the least troublesome and cheapest manner possible. We may pass as generous in our own estimation for the twopence we give to the beggar, and go on our way with the pleasant feeling of self-satisfaction, which is cheaply bought at the price. We could not offer the same twopence to the starving father of a family, were we to enquire into the case, nor could we give it to the district visitor to distribute; we should feel very mean if we did, and so we some of us are indebted to the beggar on the whole. We do not want to realise poverty and misery; to contrast want with our own superfluity, or sickness with our abundant health. The beggar lets us dismiss unpleasantness for the lordly sum of twopence, and even gives us something in exchange. Only now and then are our consciences disturbed by these inopportune proofs that our charity is not all-sufficient after all. But the pity dies to-morrow, the beggar gains by the idle life that pays, whilst honest poverty, with pride that will not ask, lies dying on our doorstep.

It has been decided by the Committee of the Great Northern Central Hospital to erect the additional wing, which will complete the buildings, as a memorial to the late president of the institution, H.R.H. the Duke of Clarence and Avondale. Tenders for the work have been accepted, the cost of which, with the necessary fittings, will amount to about £30,000. Few events in the hospital world should excite more interest than this decision, seeing that with the completion of the new wing it will be possible to test the scientific bearings of the relative advantages and disadvantages of circular and rectangular wards. The plans have been so settled, that it will be possible at the Great Northern Central Hospital to work out this problem on identical lines, and so to supply authoritative evidence of the special value to be attached to either or both of these systems of construction.

Very recent testimony proves that the Chinese indifference to life shows itself as strong as ever in many ways. The objection to a large female population is demonstrated in the same way, and thousands of infants of the female sex are destroyed remorselessly every year. We read with horror of the melancholy part played by the Ganges in infanticide in India, but, after all, the mighty river which carries the traces of the dark deeds away to the ocean suggests far less brutality than the towers scattered over the districts of China. These buildings are literal monuments of crime. They are about thirty feet high, but have a hole in the wall reaching into the pit formed by the hollow centre of the tower. Through the hole the infants are dropped into the pit, which contains quicklime, which soon consumes the bodies. It is said that over 200,000 female children are slaughtered every year in China. There is a female orphanage at Hankow which has played an important part in rescuing these children, frequently abandoned to death, when active means are not resorted to. It has saved over 30,000 children during the twenty years of its existence.

This is an age of rampant pessimism, and so the best efforts of the best men are often cruelly criticised by the impotent or indolent. Yet there never was a time when the need for personal service in all directions was greater, and certainly, so far as our hospitals are concerned, the absence of this personal service to a much greater extent than it is at present forthcoming, is a marked defect of the present system. Yet it cannot be doubted that there are as many true hearts and willing hands available now as ever, if only they could be attached to the cause. A pathetic little story reaches us from a London suburb. A poor curate who had been much indebted to the services of his medical man, had often wished to show his gratitude in some practical form. In due course the doctor was seized with a most painful malady, which deprived him of all power of sleep, owing mainly to the agony which unnerved him. The curate devoted himself to his medical friend, and one night after the trains had ceased running the doctor, in his sufferings, declared that if he could get a particular drug relief must follow its administration. The curate took his hat, and walked some eight miles into the heart of London, secured the drug, walked back again, and brought it to his friend, who was greatly benefited by the new treatment. Personal service of this kind is the salt of the earth, and we have no doubt that the curate has his reward, although he sacrificed his night's rest in the cause of his friend.

It is really refreshing to turn to such an example of local spirit as is presented in the new Convalescent Home for Children, Moseley Hall, at Birmingham. The *Birmingham Gazette* makes its appeal with a happy confidence which shows an assurance of a successful result. "Beds, mattresses, bedding, and guineas—especially guineas—are demanded on behalf of Moseley Hall." The Old Manor House has been given by Mr, Richard Cadbury. It stands in a garden of twenty-two acres, an undoubted source of delight to the small inmates. Cots, mattresses, perambulators, and all kinds of essentials—toys not being forgotten—have come from the hands of numerous kind friends. Matron and staff are already established, and even one patient has been received, although the Home only professes to be ready this week. Fifty little ones can be taken in when all is completed. It is estimated that the cost per bed will amount to £20 per annum, and it is hoped that subscriptions will permit a larger admission as time goes on. Until now Birmingham has had no convalescent home for children, and the opportunity here given to child-patients in the hospitals will, no doubt, be eagerly sought for; blankets and guineas will, it is to be hoped, be as easily forthcoming.

---

China of to-day is not so very different to the China of yesterday. For the spirit of progress is not the ruling force there as with the little neighbour, Japan. Not long ago a correspondent supplied us with facts strange and horrible, revealing the absence of all attempts to battle with disease and mitigate the sufferings of the teeming population. The first point that strikes us, said he, in our walks abroad, is the terrible amount of walking disease. Disease of all kinds and in all stages parades the highways. Why trouble about that insignificant factor, life? Life is the cheapest of all commodities in China. There is so much of it. Even a malefactor can buy a substitute for a few hundred dollars. Fractures and diseases are mostly left to chance. As for the science of surgery, there is practically none. The Chinese have no surgical instruments. Our correspondent tells us of one man he met who, having lost one foot, was walking about on the bare bone, the flesh having receded several inches, and was in a sore condition, upon which the flies had settled. Deformity, indeed, provides a living for the afflicted, and it is said that many female children are blinded intentionally. No one may turn a beggar from their door unless provided with food, and so the trade of begging and deformity goes on unchecked. The Chinese are not without some knowledge of the use of drugs, which appear to be of a vegetable nature. In the expedition of 1858-61. the surgeons of the force who established a small hospital near Canton, adopted some of the fever remedies, which proved very efficacious. A curious method of curing sunstroke is said to have good effect. The flesh on both sides of the back-bone is squeezed between two coins, until a red ridge is formed, which causes, so it is said, a cure. Dentistry is in the hands of the barbers, who are very skilful in extracting, but this is the limit of their art. The chiropodists show more method, and effect good cures. A gradual belief in the superiority of European methods is spreading, and European doctors are sometimes employed by the more enlightened. In one case doctors were called in too late to attend a high official suffering from abscess of the liver. He had undergone a treatment of ground elephant's tusks. Time and experience will pave the way to medical knowledge, and with it these terrible exhibitions of disease will cease in Chinese highways.

